# Conductivity and radio frequency performance data for silver nanoparticle inks deposited via aerosol jet deposition and processed under varying conditions

**DOI:** 10.1016/j.dib.2020.106331

**Published:** 2020-09-24

**Authors:** James R. Deneault, Carrie Bartsch, Alexander Cook, Christopher Grabowski, J. Daniel Berrigan, Nicholas Glavin, Philip R. Buskohl

**Affiliations:** a Air Force Research Laboratory, Wright-Patterson AFB, OH, 45433, USA; b Universal Technology Company, Beavercreek, OH, 45432, USA; c UES, Inc., Dayton, OH, 45432, USA

**Keywords:** Aerosol jet printing, Silver nano-particle ink, Conductivity, Co-planar waveguide, Radio frequency, Sintering, Insertion loss

## Abstract

In fabricating electronic components or devices via Aerosol Jet Printing (AJP) there are numerous options for commercially available Metal NanoParticle (MNP) inks. Regardless of the MNP ink selected, the electrical properties of the final product are not commensurate to those of the bulk metal due to the inherent porosity and impurity-infused composition that is characteristic of these heterogeneous feedstock. Hence, choosing the best MNP ink for a particular application can be difficult, even among those based on the same metal, as each ink formulation can yield different performance metrics depending on the specific formulation and the conditions under which it is processed. In this article, the DC conductivity of AJP pads and the Radio Frequency (RF) transmission loss of AJP Coplanar Waveguides (CPWs) are presented for three different, commercially available silver MNP inks; Advanced Nano Products (ANP) Silverjet DGP 40LT-15C, Clariant Prelect TPS 50 G2, and UT Dots UTDAg40X. We determined conductivity values by measuring the printed pad thicknesses using stylus profilometry and measuring sheet resistances using a co-linear 4-point probe. Additionally, we collected RF spectra using a performance network analyzer over the 10 MHz – 40 GHz range. A complete description of the preparation, AJP procedure, and sintering is provided. Conductivity and RF data are presented for several scenarios including sintering temperatures, sintering atmospheres, and un-sintered storage conditions. We anticipate this dataset will serve as a useful reference for benchmarking electrical performance and troubleshooting pre- and post-processing steps for Ag nanoparticle based AJP inks.

## Specifications Table

**Table:** 

Subject	Materials Science (General)
Specific subject area	Additive manufacturing of electrical components and devices by aerosol jet deposition
Type of data	Raw, Tables, Graphs, and Images
How data were acquired	Co-linear 4-point probe measurements: Signatone SP4-40045TBJ, Signatone S-725SRM, Signatone L-4PQM, Keithley 2400 Source Meter, LabVIEW Stylus Profilometry: Bruker Dektak XT Sintering: Fisher Scientific Isotemp 282A, Type K Thermocouple (Nickel Probe), MF52C1103F3380 Thermistor, Keithley 2400 Source Meter, LabVIEW RF Analysis: Keysight E8364B PNA, Cascade manual/semi-automatic probe station, 250 micron pitch Cascade Air Coplanar Probes (ACP),
Data format	Raw data text files and post-processed data in tables and graphs
Parameters for data collection	DC conductivity and RF spectra were measured as a function of sample sintering temperature and atmosphere. The sintering temperatures examined were 145°C, 165°C, 185°C, 205°C, and 225°C. The sintering atmospheres chosen were air, nitrogen, and vacuum. Un-sintered sample storage atmospheres were air and vacuum and ranged from 0 to 8 days.
Description of data collection	Samples were fabricated using an Optomec Aerosol Jet Deposition System (AJ300-UP) with a Sprint UA Max Ultrasonic Atomizer to produce the aerosol. We chose to investigate three commercially available silver nano-particle inks; ANP Silverjet DGP 40LT-15C, Clariant Prelect TPS 50 G2, and UT Dots UTDAg40x. DC conductivity measurements were acquired by taking the reciprocal of the product of measured sheet resistance and sample thickness. Sheet resistance measurements were obtained using a co-linear 4-Point Probe (4PP) connected to a source meter controlled via a LabVIEW program wherein the voltage was swept from -1 V to +1 V between the two center pins and the resulting current was measured between the two outer pins. Radio frequency measurements were taken using an on-wafer manual/semi-automated microwave probe station from Cascade in ambient air, using a Keysight E8364B PNA network analyzer having a frequency range of 10 MHz to 50 GHz. Additionally, 250 micron pitch Cascade Air Coplanar Probes (ACP) were used to measure the S parameters. Before taking measurements, the system was calibrated using an on-wafer Line-Reflect-Reflect-Match (LRRM) calibration substrate over the range of 10 MHz to 40 GHz.
	Sintering temperature profiles were recorded using an Omega Type K thermocouple probe connected to a Keithley 2400 Source Meter. As required, the values recorded using the thermocouple probe were corrected using a calibrated MF52 10k thermistor at the cold junction. Values were measured, corrected, and recorded through the source meter using a custom LabVIEW Virtual Instrument (VI). Profilometry data of 4PP pads were collected using a Bruker Dektak XT Profiler fitted with a 2 µm stylus. Individual scans were taken with a stylus force of 0.03 mg and at a scan rate of 71.16 µm/s. 10 scans were taken per pad with a raster pitch of 700 µm. The data was tilt and curvature corrected, and data flattening was performed with respect to the substrate prior to acquiring the average film thickness values.
Data source location	Air Force Research Laboratory, Wright-Patterson Air Force Base, Ohio 45433, United States
Data accessibility	The raw data is available in the supplementary data files uploaded to this manuscript submission.

## Value of the Data

• The data provides valuable insight into the electrical performance of three commercially available silver nano-particle inks as a function of sintering temperature, sintering atmosphere, and sample storage conditions.
• The data reinforces the importance of choosing the appropriate processing conditions for each ink and supplements existing guidelines for processing inks to achieve optimal results for electronics applications.
• Provides a benchmark dataset of DC and RF electrical performance to leverage in design modeling tools and for comparing print performance with other AJP inks.
• The data elucidates the performance variability between silver nanoparticle inks and sensitivity to pre- and post-processing conditions.
• Ink developers, designers and manufacturers of printed electronics, quality control engineers and others would benefit from this baseline characterization of commercial AJP ink performance across a large range of pre- and post-processing parameters.

## 1 Data Description

Raw data from several aspects of the study are provided:

• DC sheet resistance measurements
• Profilometry of deposited samples
• S-parameter (S2P) data from RF testing of the co-planar waveguides
• Temperature profiles for sample sintering

Specific experimental parameters are also provided in text file format:

• Deposition parameters and details
• Representative ACSPL+ code used for printing
• Profilometry parameters and details
• Sintering procedures
• Storage procedures

**Fig. 1:**
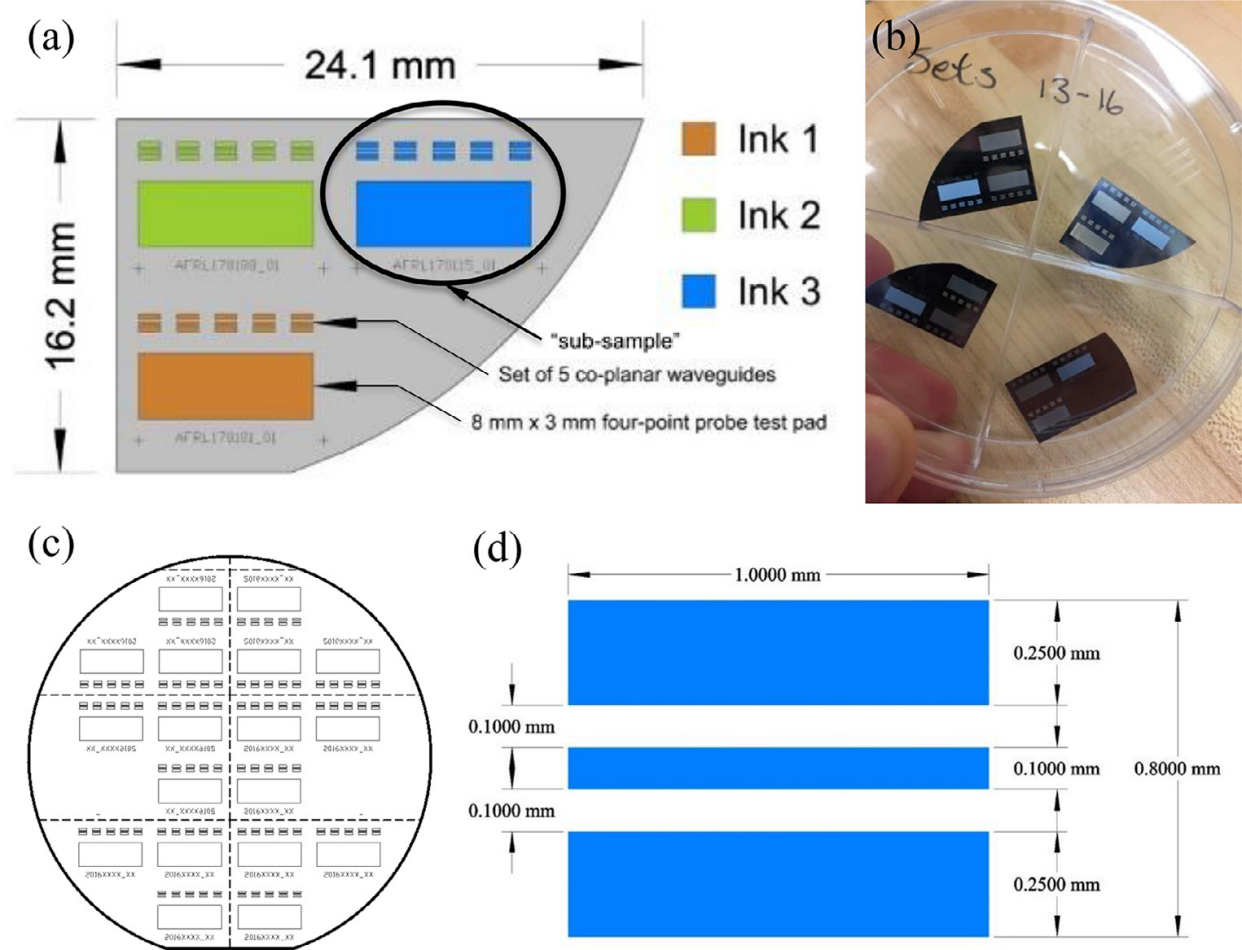
Sample layout details for the sintering study. Three sub-samples, one from each ink, are deposited on each sample substrate (a). Each sub-sample consists of five co-planar waveguides and one 4-point probe pad. Sample substrates are intrinsic silicon (280µm, >10,000 Ohm•cm) and are obtained by dicing 2 inch (50.8 mm) wafers as shown by the dashed lines in (c). The target co-planar waveguide geometry is shown in (d).

Data files can be referenced using several methods. Files corresponding to individual sub-samples (Fig. 1) can be referenced using sample names (e.g. “ANP_05_01.S2P”). Files corresponding to specific measurements can be referenced by files suffix as:

• “.ASC” corresponds to height data
• “.S2P” corresponds to radio frequency S-parameters
• “.lvm” corresponds to sintering temperature profiles
• “.prg” corresponds to ACSPL+ toolpath files

Additional raw data and specific measurement or procedural details are included in “.txt” files and are easily identified by filename (e.g. “ANP Pre-Sinter Storage Study.txt”)

Fig. 1 presents sample layout details for the co-planar waveguide sintering study.

**Fig. 2:**
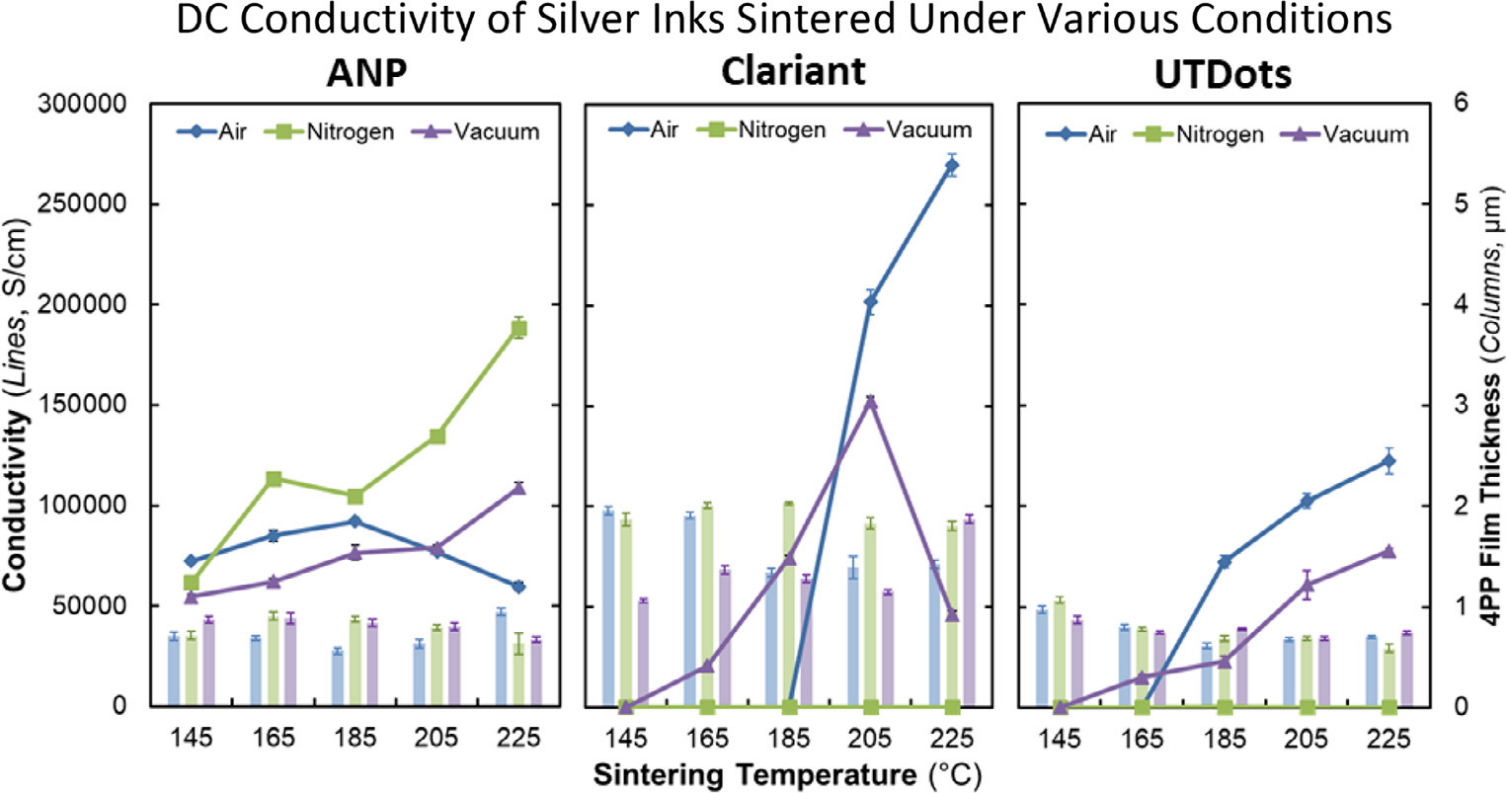
Average DC conductivity and four point probe pad thickness vs. sintering temperature for each ink in each sintering atmosphere. Standard deviations values were derived from three sheet resistance measurements per sample and measured z-height deviation over the surface of the pads (see Fig. 11). Samples were sintered using recipes that ramped to the target temperature in 1 hour, dwelled at the target temperature for ½ hour, then passively cooled to < 75°C over ∼5 hours (see Fig. 10).

Fig. 2 presents plots of average DC conductivity and 4PP pad thickness against sintering temperature for each ink in each sintering atmosphere.

**Fig. 3:**
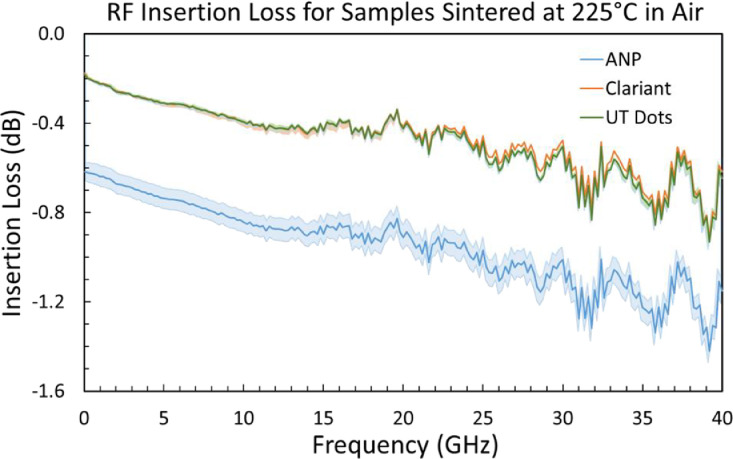
Example of average S21 (insertion loss) spectra for co-planar waveguides printed using each of the three inks and sintered at 225°C. Shaded regions represent the standard deviation for five CPWs per sample.

Fig. 3 provides example insertion loss spectra for co-planar waveguides printed using each of the three inks and sintered at 225 °C.

**Fig. 4:**
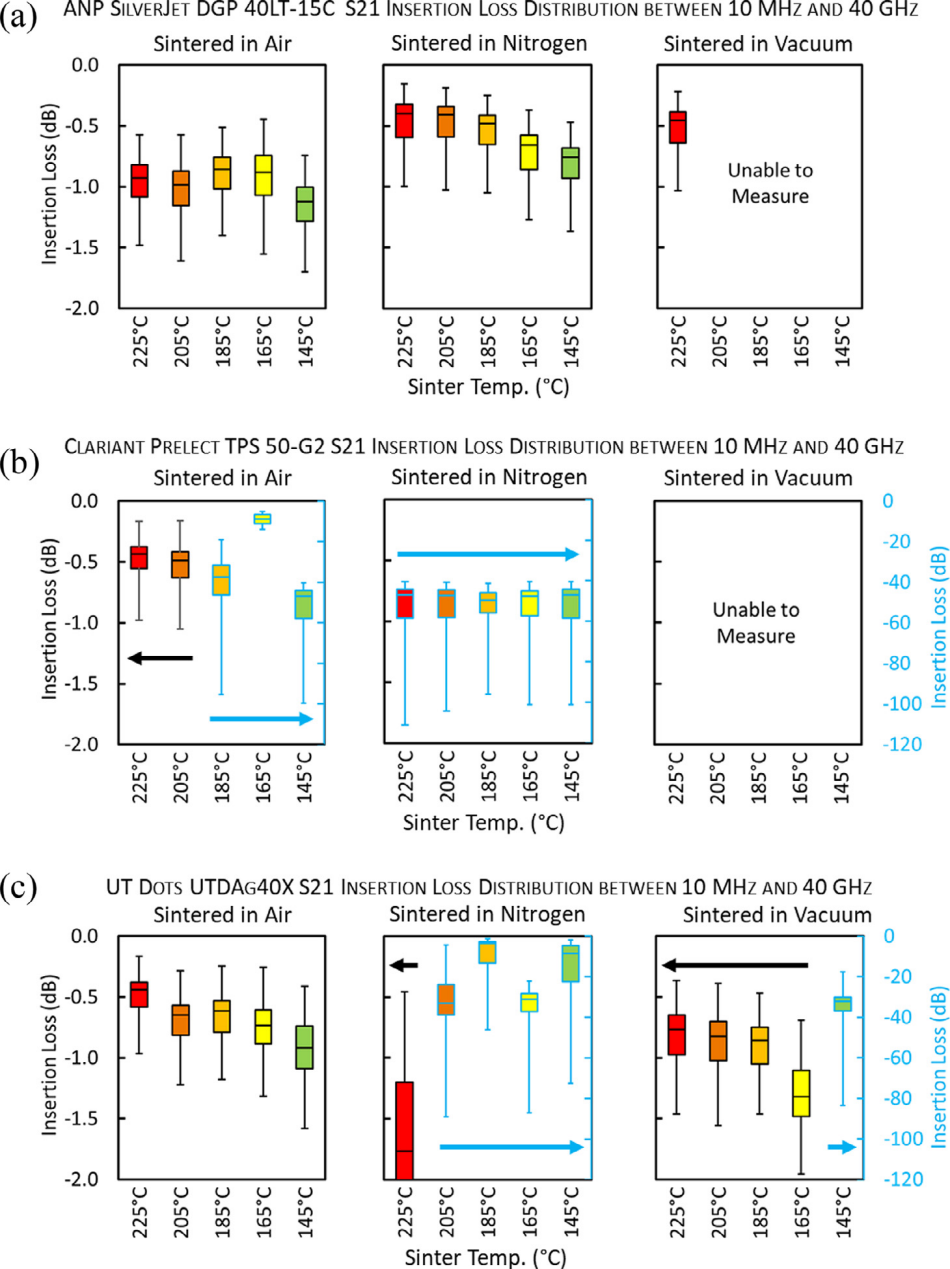
Boxplots which graphically summarize the insertion loss distribution over the 10 MHz to 40 GHz range for each of the inks sintered under each condition is shown. This graphing method depicts five key points for each sintering condition; the minimum value, the first quartile value, the mean value, the third quartile value, and the maximum value. A secondary y-axis (blue) is added to (b) and (c) to more clearly present the insertion loss distribution over two distinct regimes. Some CPWs scratched easily or delaminated when probed for RF interrogation; these were labeled as “unable to measure”.

Fig. 4. (a)-(c) employs box plots to graphically summarize the quartile insertion loss distribution over the 10 MHz to 40 GHz range for each of the inks sintered under each condition. Fig. 4. (b) and (c) contain secondary y-axes in order to more clearly present the insertion loss distribution over two distinct regimes.

**Fig. 5:**
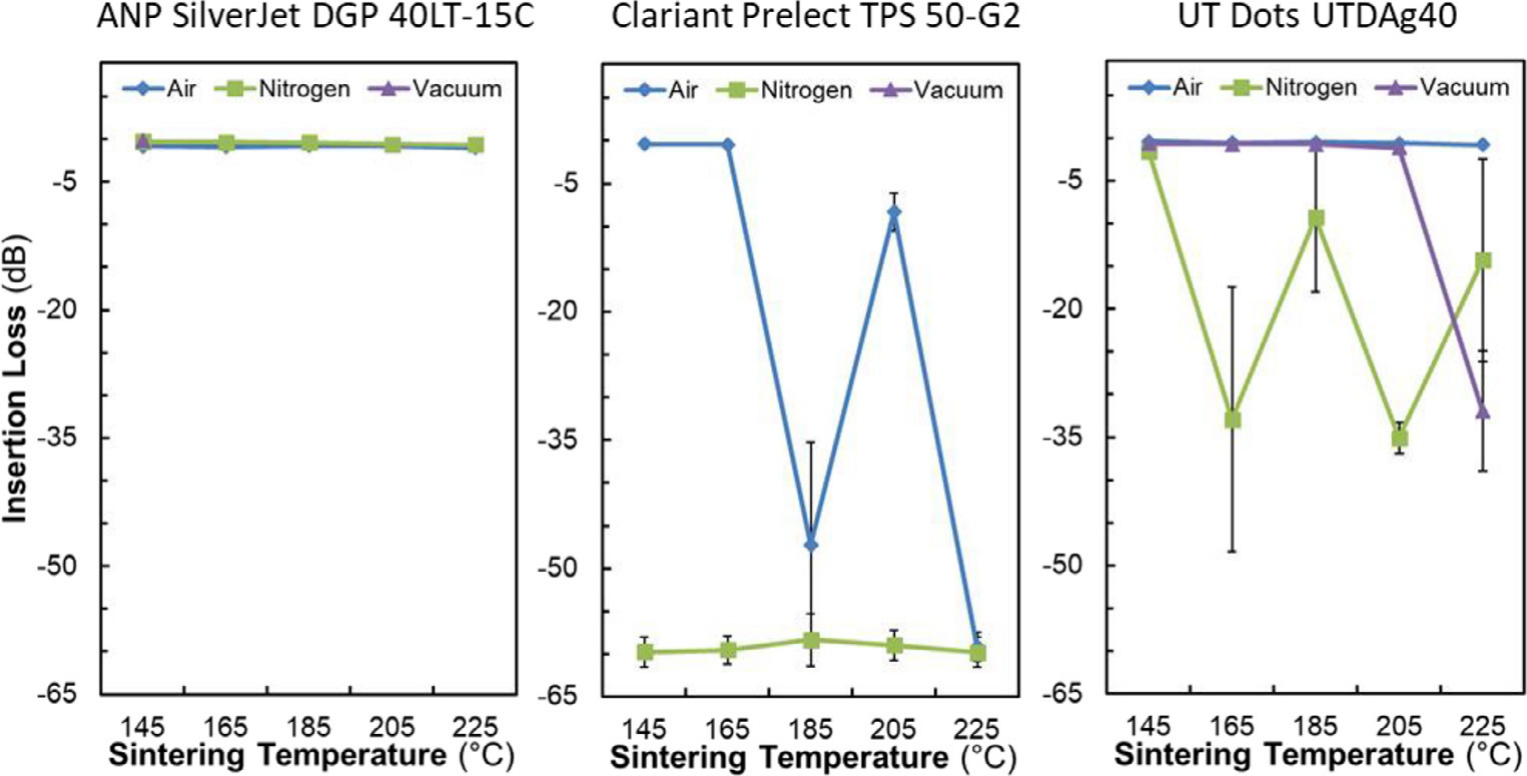
A subset of the insertion loss data (mean ± SD, n=5) is shown wherein insertion losses are averaged over the x-band range (7 GHz – 11.2 GHz) and plotted as a function of sintering temperature.

Fig. 5 presents a subset of the insertion loss data wherein insertion losses are averaged over the x-band range (7 GHz–11.2 GHz) and plotted as a function of sintering temperature.

**Fig. 6:**
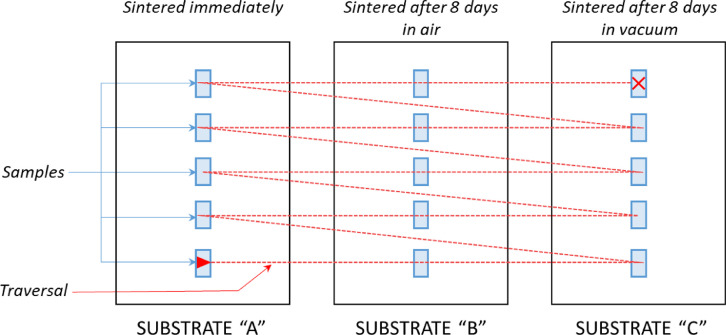
A diagram showing the scheme used for printing and comparing the storage effects for yet-to-be-sintered samples. For each of the three inks, we printed a total of fifteen 8 mm x 3 mm samples across three clean 2” x 3” glass slides using the traversal scheme indicated by the red dashed line. Immediately following deposition, we sintered substrate “A” and analyzed the pads for DC conductivity. Substrates “B” and “C” were stored in air and under vacuum, respectively, for 8 days prior to sintering and DC conductivity analysis.

Fig. 6 provides a diagram showing the scheme used for printing and comparing the storage effects for yet-to-be-sintered samples.

**Fig. 7:**
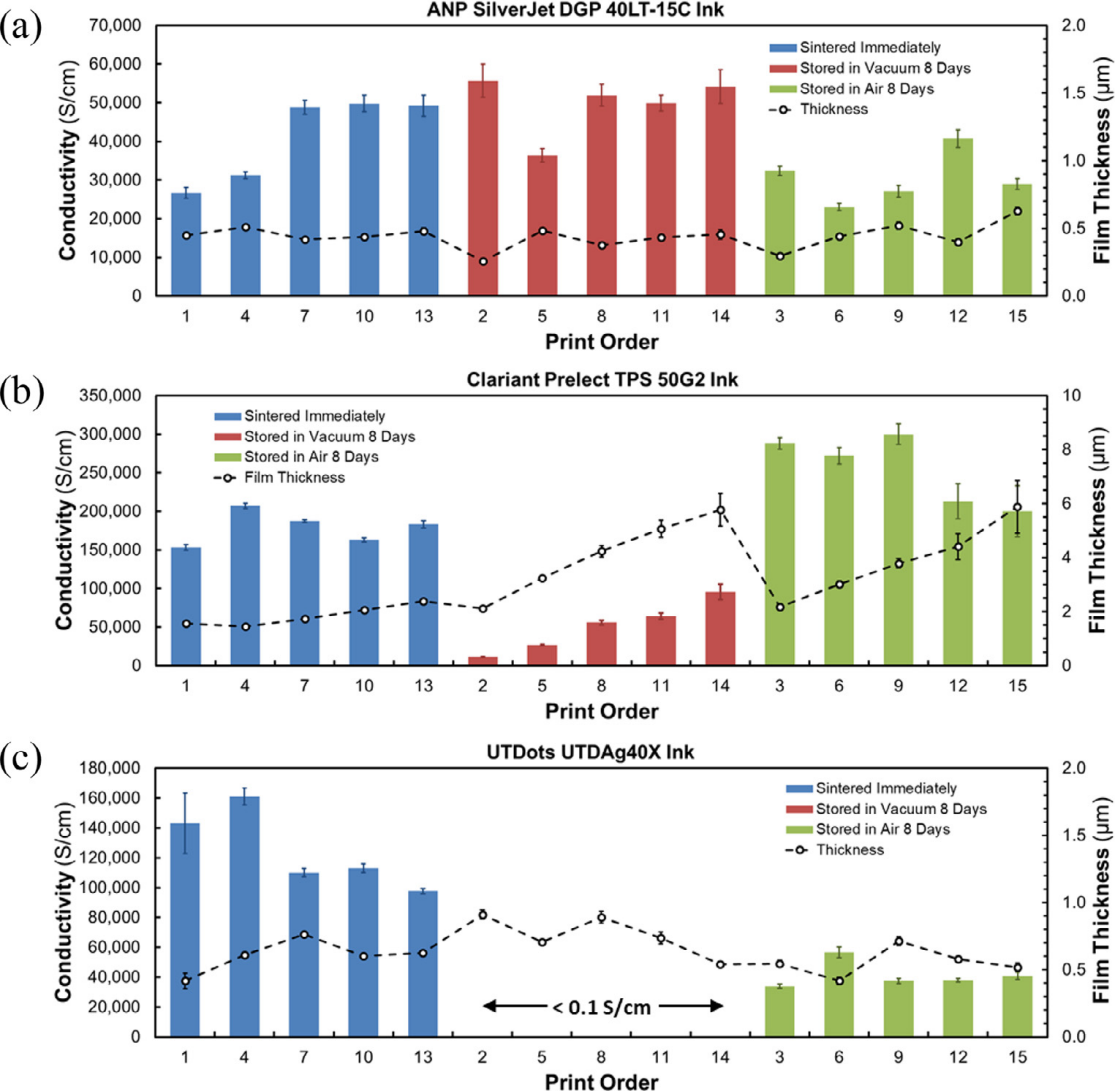
The effect of yet-to-be-sintered sample storage conditions on the post-sintering conductivity. Shown in (a) are the conductivity results and thicknesses for samples printed using ANP ink and sintered at ∼165°C for 60 minutes. Here, samples stored under vacuum for 8 days yielded the best results. The results for Clariant ink are shown in (b) where samples were sintered at ∼190°C for 60 minutes. The best conductivity results for Clariant samples were for those stored 8 days in air prior to sintering. Finally, the results for UT Dots samples are shown in (c). Here, samples were sintered at 175°C for 60 minutes and those sintered immediately after printing yielded the best conductivity results.

Fig. 7 presents data that elucidates the effects of yet-to-be-sintered sample storage conditions on the post-sintering conductivity.

**Fig. 8:**
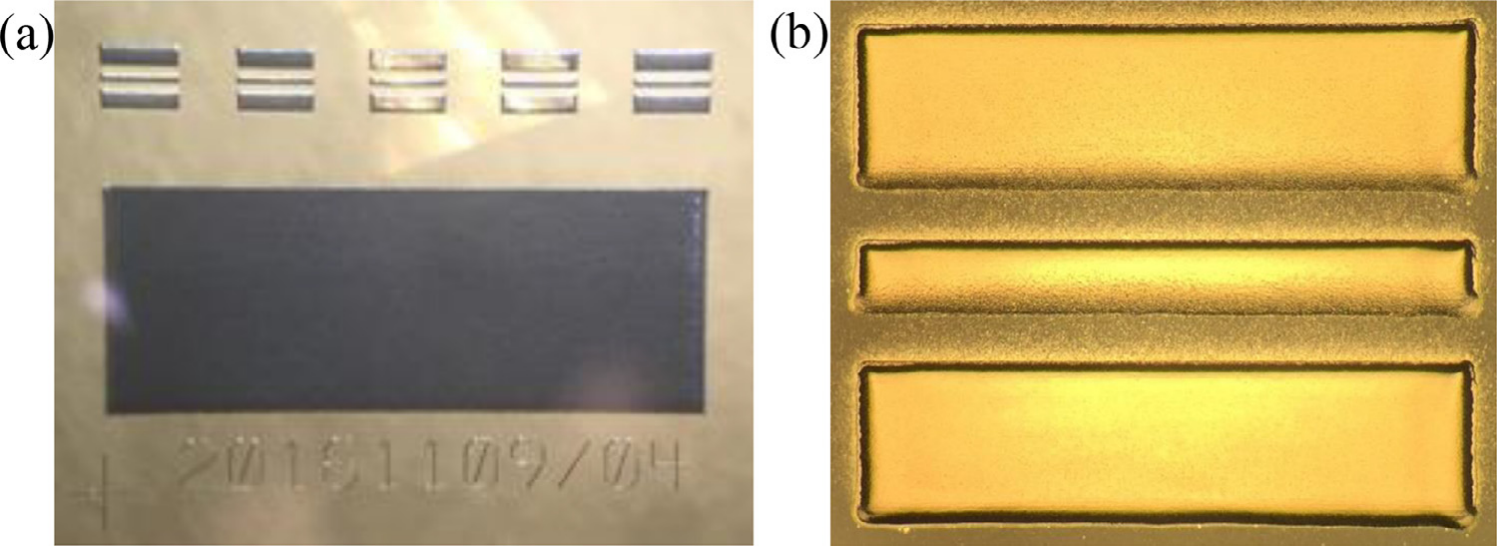
An optical micrograph of a complete sample (on glass) is shown in (a). The complete sample comprises five co-planar waveguides and a single 8 mm x 3 mm pad for 4-point probe conductivity measurements. An optical micrograph of an individual co-planar waveguide (on silicon) is shown in (b). The waveguides are 1 mm wide, have a 100 µm wide center conductor, and gap widths of 100 µm.

Fig. 8 presents a representative optical micrograph of the as-printed pad for 4-pt probe measurements and the co-planar wavegude geometries used for characterizing the electrical performance of the Ag nanoparticle inks in this study.

**Fig. 9:**
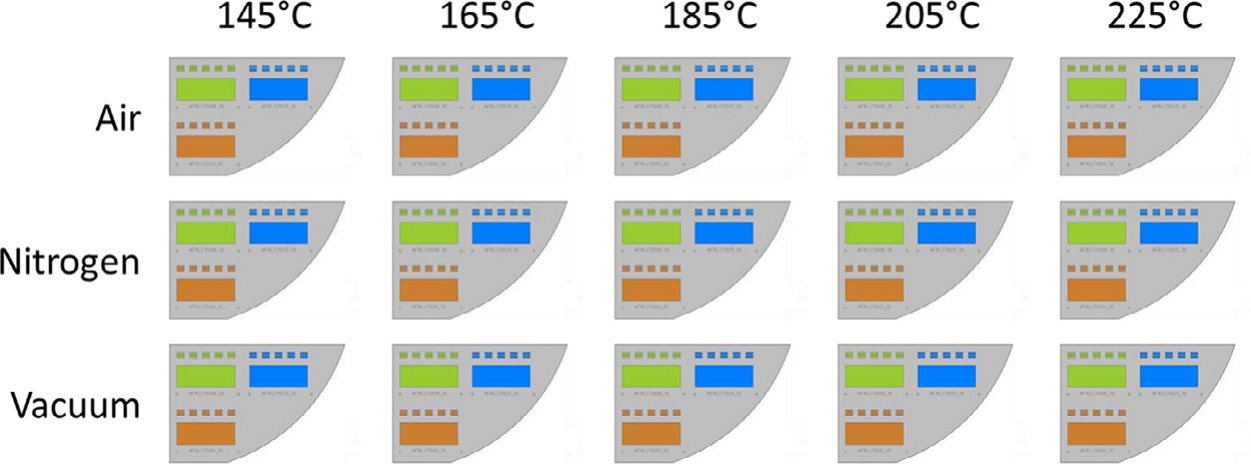
A graphical layout of the sintering scheme. Each substrate contains a sub-sample printed from each of the three inks, indicated by color.

Fig. 9 presents a schematic of the printing arrangement on the substrate for all specimens used in the sintering study.

**Fig. 10:**
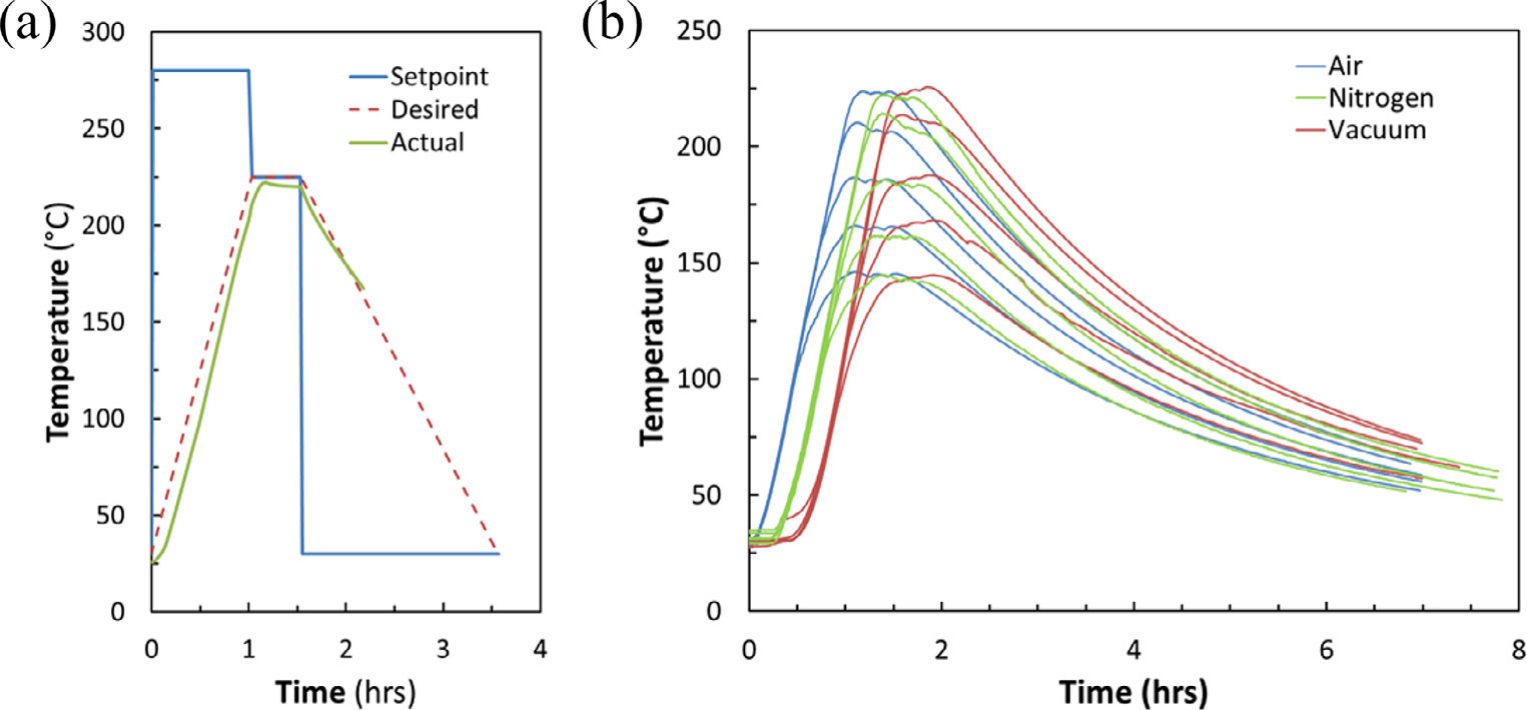
An example of a custom sintering program where a desired temperature of 225°C for 30 minutes was targeted is shown in (a). Actual sintering temperature profiles for all fifteen substrates are shown in (b). Here, the samples cool passively for approximately 5 hours before being removed from the oven.

Fig. 10 presents representative temperature vs time profiles used for sintering the Ag nanoparticle inks of this study.

**Fig. 11:**
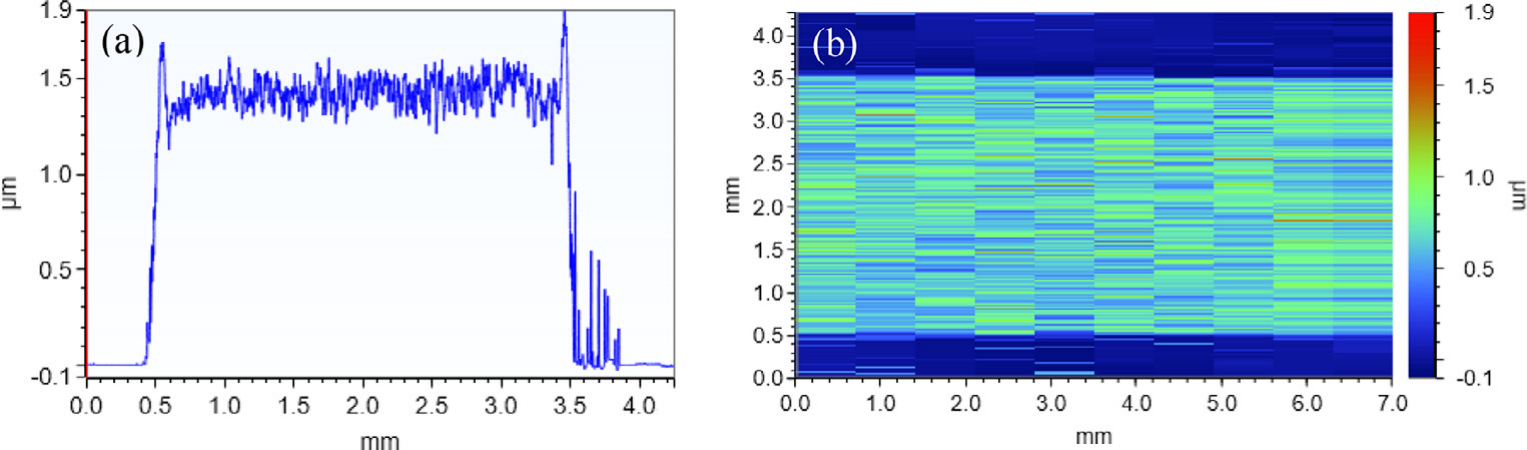
Representative profilometry data is shown. A single 2D profile across the width of a sub-sample's 4-point probe pad is shown in (a). A 3D map of a 4-point probe pad is shown in (b). Here, ten 2D profiles are combined in a top-down view where the height values are shown using the calibrated color scale shown on the right.

Fig. 11 contains a representative 2D and 3D profilometry dataset, indicating how the thickness measurement was performed for each specimen.

Table 1 presents a summary dataset of electrical performance for aerosol jet printed specimens using ANP SilverJet DGP 40LT-15C ink under various sintering conditions

**Table 1:** Summary data for aerosol jet printed specimens using ANP SilverJet DGP 40LT-15C ink under various sintering conditions.

Sintering Study Summary for ANP SilverJet DGP 40LT-15C						
Sample	Air Storage Duration (days)	Sintering Atmo- sphere	Sintering Temp- erature (°C)	Conductivity (S/cm)†	Film Thickness (µm) †	Average Transmission loss (S21, dB) †
ANP_01	10	Air	225	59708 ± 2143	0.941 ± 0.040	-0.95 ± 0.18
ANP_02	10	Air	205	77010 ± 1240	0.625 ± 0.039	-1.01 ± 0.21
ANP_03	11	Air	185	92056 ± 1818	0.553 ± 0.031	-0.88 ± 0.19
ANP_04	11	Air	165	85007 ± 2759	0.680 ± 0.020	-0.91 ± 0.23
ANP_05	14	Air	145	72351 ± 1025	0.701 ± 0.042	-1.15 ± 0.19
ANP_06	15	Nitrogen	225	188655 ± 5431	0.626 ± 0.109	-0.45 ± 0.18
ANP_07	17	Nitrogen	205	134678 ± 1727	0.788 ± 0.029	-0.46 ± 0.17
ANP_08	18	Nitrogen	185	104875 ± 2896	0.869 ± 0.026	-0.53 ± 0.17
ANP_09	17	Nitrogen	165	113488 ± 895	0.900 ± 0.039	-0.71 ± 0.19
ANP_10	15	Nitrogen	145	61695 ± 1391	0.707 ± 0.045	-0.80 ± 0.19
ANP_11	18	Vacuum	225	108705 ± 2883	0.665 ± 0.029	-0.51 ± 0.17
ANP_12	21	Vacuum	205	79067 ± 1061	0.793 ± 0.035	- ± -
ANP_13	22	Vacuum	185	76484 ± 3556	0.832 ± 0.037	- ± -
ANP_14	22	Vacuum	165	62146 ± 1606	0.880 ± 0.054	- ± -
ANP_15	21	Vacuum	145	54638 ± 1165	0.867 ± 0.031	- ± -

† mean ± SD. * “-” denotes failed measurements due to scratching or delamination.

Table 2 presents a summary dataset of electrical performance for aerosol jet printed specimens using Clariant Prelect TPS 50-G2 ink under various sintering conditions.

**Table 2:** Summary data for aerosol jet printed specimens using Clariant Prelect TPS 50-G2 ink under various sintering conditions.

Sintering Study Summary for Clariant Prelect TPS 50-G						
Sample	Air Storage Duration (days)	Sintering Atmo- sphere	Sintering Temp- erature (°C)	Conductivity (S/cm) †	Film Thickness (µm) †	Average Transmission loss (S21, dB) †
CLA_01	16	Air	225	269947 ± 5623	1.426 ± 0.040	-0.47 ± 0.15
CLA_02	16	Air	205	201831 ± 6206	1.395 ± 0.111	-0.52 ± 0.17
CLA_03	17	Air	185	42 ± 6	1.343 ± 0.039	-39.53 ± 13.12
CLA_04	17	Air	165	0 ± 0	1.911 ± 0.031	-9.02 ± 2.60
CLA_05	19	Air	145	0 ± 0	1.958 ± 0.039	-52.05 ± 10.88
CLA_06	20	Nitrogen	225	0 ± 0	1.803 ± 0.046	-52.34 ± 11.14
CLA_07	22	Nitrogen	205	0 ± 0	1.832 ± 0.053	-52.36 ± 10.74
CLA_08	23	Nitrogen	185	0 ± 0	2.031 ± 0.017	-52.49 ± 9.49
CLA_09	22	Nitrogen	165	0 ± 0	2.005 ± 0.029	-52.25 ± 10.41
CLA_10	20	Nitrogen	145	0 ± 0	1.870 ± 0.061	-52.39 ± 11.16
CLA_11	23	Vacuum	225	46176 ± 1682	1.874 ± 0.039	- ± -
CLA_12	26	Vacuum	205	152397 ± 1995	1.149 ± 0.023	- ± -
CLA_13	27	Vacuum	185	74239 ± 1523	1.282 ± 0.035	- ± -
CLA_14	27	Vacuum	165	20729 ± 508	1.371 ± 0.039	- ± -
CLA_15	26	Vacuum	145	0 ± 0	1.060 ± 0.017	- ± -

† mean ± SD. * “-” denotes failed measurements due to scratching or delamination.

Table 3 presents a summary dataset of electrical performance for aerosol jet printed specimens using UT Dots UTDAg40x ink under various sintering conditions.

**Table 3:** Summary data for aerosol jet printed specimens using UT Dots UTDAg40x ink under various sintering conditions.

Sintering Study Summary for UT Dots UTDAg40						
Sample	Air Storage Duration (days)	Sintering Atmo- sphere	Sintering Temp- erature (°C)	Conductivity (S/cm) †	Film Thickness (µm) †	Average Transmission loss (S21, dB) †
UTD_01	2	Air	225	122522 ± 6531	0.701 ± 0.016	-0.48 ± 0.16
UTD_02	2	Air	205	102534 ± 3834	0.674 ± 0.019	-0.68 ± 0.19
UTD_03	3	Air	185	72352 ± 2922	0.610 ± 0.028	-0.65 ± 0.19
UTD_04	3	Air	165	0 ± 0	0.797 ± 0.027	-0.75 ± 0.20
UTD_05	6	Air	145	0 ± 0	0.976 ± 0.038	-0.92 ± 0.23
UTD_06	7	Nitrogen	225	0 ± 0	0.589 ± 0.043	-2.05 ± 1.15
UTD_07	8	Nitrogen	205	0 ± 0	0.688 ± 0.020	-30.92 ± 16.15
UTD_08	9	Nitrogen	185	192 ± 301	0.684 ± 0.027	-8.30 ± 8.02
UTD_09	8	Nitrogen	165	0 ± 0	0.775 ± 0.018	-34.14 ± 10.11
UTD_10	7	Nitrogen	145	0 ± 0	1.067 ± 0.032	-13.52 ± 11.85
UTD_11	9	Vacuum	225	77880 ± 1349	0.739 ± 0.018	-0.80 ± 0.22
UTD_12	12	Vacuum	205	60864 ± 7241	0.686 ± 0.017	-0.86 ± 0.23
UTD_13	13	Vacuum	185	22826 ± 2634	0.778 ± 0.010	-0.89 ± 0.20
UTD_14	13	Vacuum	165	15141 ± 958	0.745 ± 0.012	-1.31 ± 0.26
UTD_15	12	Vacuum	145	0 ± 0	0.874 ± 0.038	-32.78 ± 10.44

† mean ± SD.

Table 4 presents a summary dataset of conductivity for aerosol jet printed specimens using ANP SilverJet DGP 40LT-15C ink sintered after storing under various conditions.

**Table 4:** Summary data for aerosol jet printed specimens using ANP SilverJet DGP 40LT-15C ink sintered after storing under various conditions.

Storage Study Summary for ANP SilverJet DGP 40LT-15C				
Sample	Storage Condition	Sintering Temperature (°C)†	Conductivity (S/cm)‡	Film Thickness (µm)‡
ANP_S_01	No storage; sintered Immediately	165.5 ± 4.5	26700 ± 1400	0.4487 ± 0.0240
ANP_S_04		165.5 ± 4.5	31300 ± 900	0.5099 ± 0.0140
ANP_S_07		165.5 ± 4.5	48800 ± 1800	0.4177 ± 0.0151
ANP_S_10		165.5 ± 4.5	49800 ± 2200	0.4360 ± 0.0191
ANP_S_13		165.5 ± 4.5	49200 ± 2800	0.4785 ± 0.0270
ANP_S_02	Stored in vacuum for 8 days before sintering	164.0 ± 4.0	55700 ± 4300	0.2589 ± 0.0201
ANP_S_05		164.0 ± 4.0	36400 ± 1700	0.4847 ± 0.0226
ANP_S_08		164.0 ± 4.0	51900 ± 2800	0.3768 ± 0.0204
ANP_S_11		164.0 ± 4.0	49900 ± 2100	0.4341 ± 0.0179
ANP_S_14		164.0 ± 4.0	54200 ± 4300	0.4556 ± 0.0364
ANP_S_03	Stored in air for 8 days before sintering	164.0 ± 4.0	32400 ± 1300	0.2970 ± 0.0115
ANP_S_06		164.0 ± 4.0	23000 ± 1000	0.4408 ± 0.0188
ANP_S_09		164.0 ± 4.0	27100 ± 1500	0.5202 ± 0.0297
ANP_S_12		164.0 ± 4.0	40700 ± 2200	0.3982 ± 0.0218
ANP_S_15		164.0 ± 4.0	29000 ± 1400	0.6281 ± 0.0302

† mean ± avg. dev.

‡ mean ± SD

Table 5 presents a summary dataset of conductivity for aerosol jet printed specimens using Clariant Prelect TPS 50-G2 ink sintered after storing under various conditions.

**Table 5:** Summary data for aerosol jet printed specimens using Clariant Prelect TPS 50-G2 ink sintered after storing under various conditions.

Storage Study Summary for Clariant Prelect TPS 50-G2				
Sample	Storage Condition	Sintering Temperature (°C)†	Conductivity (S/cm)‡	Film Thickness (µm)‡
CLA_S_01	No storage; sintered Immediately	181.5 ± 11.5	153000 ± 4000	1.5687 ± 0.0375
CLA_S_04		181.5 ± 11.5	207000 ± 3000	1.4555 ± 0.0226
CLA_S_07		181.5 ± 11.5	187000 ± 2000	1.7425 ± 0.0150
CLA_S_10		181.5 ± 11.5	163000 ± 3000	2.0624 ± 0.0334
CLA_S_13		181.5 ± 11.5	183000 ± 5000	2.3856 ± 0.0620
CLA_S_02	Stored in vacuum for 8 days before sintering	180.0 ± 12.0	11000 ± 100	2.1259 ± 0.0245
CLA_S_05		180.0 ± 12.0	26900 ± 600	3.2388 ± 0.0772
CLA_S_08		180.0 ± 12.0	55700 ± 2800	4.2312 ± 0.2095
CLA_S_11		180.0 ± 12.0	64400 ± 4000	5.0569 ± 0.3154
CLA_S_14		180.0 ± 12.0	95400 ± 10000	5.7762 ± 0.6062
CLA_S_03	Stored in air for 8 days before sintering	180.0 ± 12.0	288000 ± 7000	2.1655 ± 0.0532
CLA_S_06		180.0 ± 12.0	272000 ± 11000	3.0110 ± 0.1213
CLA_S_09		180.0 ± 12.0	300000 ± 13000	3.7889 ± 0.1703
CLA_S_12		180.0 ± 12.0	213000 ± 23000	4.4054 ± 0.4733
CLA_S_15		180.0 ± 12.0	200000 ± 33000	5.8804 ± 0.9715

† mean ± avg. dev.

‡ mean ± SD

Table 6 presents a summary dataset of conductivity for aerosol jet printed specimens using UT Dots UTDAg40x ink sintered after storing under various conditions.

**Table 6:** Summary data for aerosol jet printed specimens using UT Dots UTDAg40x ink sintered after storing under various conditions.

Storage Study Summary for UT Dots UTDAg40x				
Sample	Storage Condition	Sintering Temperature (°C)†	Conductivity (S/cm)‡	Film Thickness (µm)‡
UTD_S_01	No storage; sintered Immediately	177.0 ± 0.5	143000 ± 20000	0.4159 ± 0.0587
UTD_S_04		177.0 ± 0.5	161000 ± 6000	0.6101 ± 0.0216
UTD_S_07		177.0 ± 0.5	110000 ± 3000	0.7617 ± 0.0193
UTD_S_10		177.0 ± 0.5	113000 ± 3000	0.6021 ± 0.0163
UTD_S_13		177.0 ± 0.5	97700 ± 1700	0.6262 ± 0.0111
UTD_S_02	Stored in vacuum for 8 days before sintering	168.0 ± 8.0	0.0286 ± 0.0010	0.911 ± 0.033
UTD_S_05		168.0 ± 8.0	0.0369 ± 0.0007	0.705 ± 0.013
UTD_S_08		168.0 ± 8.0	0.0292 ± 0.0014	0.890 ± 0.043
UTD_S_11		168.0 ± 8.0	0.0360 ± 0.0022	0.736 ± 0.045
UTD_S_14		168.0 ± 8.0	0.0130 ± 0.0005	0.542 ± 0.019
UTD_S_03	Stored in air for 8 days before sintering	168.0 ± 8.0	33800 ± 1400	0.545 ± 0.023
UTD_S_06		168.0 ± 8.0	56600 ± 3700	0.416 ± 0.027
UTD_S_09		168.0 ± 8.0	37500 ± 1700	0.712 ± 0.032
UTD_S_12		168.0 ± 8.0	37900 ± 1400	0.580 ± 0.021
UTD_S_15		168.0 ± 8.0	40800 ± 2400	0.519 ± 0.030

† mean ± avg. dev.

‡ mean ± SD

Table 7 summarizes the printing parameters used for the fabrication of all specimen types for each of the Ag nanoparticle inks used in this study.

**Table 7:** A summary of general print parameters for features printed using each of the three inks. The nozzle size refers to the nominal inner diameter of the nozzle. Pressure stabilization time is the time given for the atomizer and sheath pressures to stabilize after initiating gas flows. We established the nominal trace widths by varying the sheath flow rate, the atomizer flow rate, and the print speed and measured the traces using the calibrated machine vision video feed. The print pitch refers to the pitch of the raster pattern used to print the various shapes that made up a printed feature. For features that were produced in a single coat, we employed a horizontal (i.e. lengthwise) raster pattern. For those produced using two coats, we employed a horizontal raster pattern followed by a vertical raster pattern. The approximate print time does not include the pressure stabilization time.

Ink	Feature	Nozzle Size (µm)	Pressure Stabilization Time (s)	Print Speed (mm/s)	Nominal Trace Width (mm)	Print Pitch (mm)	Number of Coats	Deposition Rate (mm³/s)	Approx. Print Time (minutes)
*ANP*	5 Co-Planar Waveguides	100	200	3	0.010	0.003	1	2.1E-05	7
	1 4-Point Probe Pad	300	30	4	0.060	0.020	2	∼3.7E-04	12
*Clariant*	5 Co-Planar Waveguides	100	200	2	0.016	0.008	1	4.6E-05	6
	1 4-Point Probe Pad	300	30	4	0.060	0.030	2	3.1E-04	8
*UT Dots*	5 Co-Planar Waveguides	100	250	3	0.030	0.009	1	†	-
	1 4-Point Probe Pad	300	30	4	0.060	0.020	2	∼4.0E-04	-

† unable to adequately fill inkwells. “ - ” indicates data unavailable.

### 1.1 Sintering study

The primary focus of this work is to elucidate the effects of sintering temperature and sintering atmosphere on the DC conductivity and RF performance of commercially available Silver Nano-Particle (AgNP) inks deposited via Aerosol Jet Printing (AJP). We chose to study **ANP SilverJet DGP 40-LT, Clariant Prelect TPS 50-G2**, and **UT Dots UTDAg40x** for this work. Tables Table 1, Table 2, Table 3 provide summarized results detailing the average conductivity (“Conductivity Data <ink>.txt” files) and average insertion loss (“.S2P” files) for each of the three inks as related to the sintering temperature and sintering atmosphere. We chose sintering temperatures of 145 °C, 165 °C, 185 °C, 205 °C, and 225 °C (“.lvm” files) and sintering atmospheres of air, nitrogen, and vacuum. The tables also include the storage duration (in air), and the average thickness of each 4-Point Probe (4PP) test pad (“.ASC” files). We obtained average insertion loss values using five separate measurements per sample (five co-planar waveguides each, Fig. 1a), and we include all measured values between 10 MHz and 40 GHz.

### 1.2 Storage study

Tables Table 4, Table 5, Table 6 show summarized results of the post-sintering DC conductivity performance of the same three AJP AgNP inks with respect to their pre-sintered storage conditions. Additional data is provided in the repository. In brief, for each of the three inks we printed a total of fifteen 8 mm × 3 mm samples across three clean 2” × 3” glass slides (Fig. 6). For each set of three substrates, we sintered one substrate immediately and stored the other two under vacuum and in air, respectively, for 8 days prior to sintering. Immediately after sintering, we measured the sheet resistances and film thicknesses in order to determine the conductivities of the samples. The results of this study are presented in Fig. 7 where conductivity values are plotted in groups corresponding to storage condition. The order in which samples were printed is also shown, as are the respective film thicknesses and sintering temperatures.

## 2 Experimental Design, Materials, and Methods

### 2.1 Sintering study

We prepared substrates by dicing two-inch (50.8 mm) intrinsic silicon wafers (280 µm, >10,000 Ohm cm; University Wafer, ID# 2018) into six sections (Fig. 1c) each of which we manually cleaned using Micro-90 detergent, followed by rinsing with distilled water, drying with a compressed air blow gun, and treating for 10 min in an 18 Watt air plasma (Harrick PDC-32G) at approximately 100 mTorr. Printing was carried out using an Optomec Aerosol Jet Deposition System (AJ300-UP) with a Sprint UA Max Ultrasonic Atomizer to produce the aerosol. We chose to investigate three commercially available silver nano-particle inks; ANP Silverjet DGP 40LT-15C (‘ANP’), Clariant Prelect TPS 50 G2 (‘Clariant’), and UT Dots UTDAg40x (‘UT Dots’). The batch dates for the ANP, Clariant, and UT Dots inks were 2014/12/11, 2016/09/29, and 2017/03/27 respectively. We tested the DC conductivities of the sintered inks prior to performing this study and verified that they were within specifications as evidenced by the conductivity results shown in Fig. 2 and Fig. 7.

Prior to loading the inks into the printer, we chose to dilute them, as appropriate, to optimize their printability. The ANP ink we used as purchased, without dilution, and a single 0.9 mL aliquot was sufficient to print all sub-samples. We diluted the Clariant ink using a high boiling point/low boiling point co-solvent motif with ethylene glycol and deionized water at 0.75 mL Clariant, 0.25 mL ethylene glycol, and 0.25 mL water. Here, we started with a 1.25 mL aliquot of diluted ink and used it to print all sub-samples. Finally, we diluted the UT Dots ink by half with terpineol and used a 1.4 mL aliquot of the diluted ink to print all sub-samples. We chose these initial volumes based on the apparent atomization efficiency (aerosol concentration by visual inspection) of each ink.

Each sub-sample comprised a large area pad for 4-Point Probe (4PP) measurements and five Co-Planar Waveguides (CPWs) as shown in Fig. 1a and Fig. 8a. Given the relative sizes and required fidelity of the printed features that made up a sample, and to optimize the time required to print them, we chose to print the CPWs using a 100 µm nozzle and the 4PP pads using a 300 µm nozzle. An optical micrograph of an individual CPW printed on silicon can be seen in Fig. 8b. In the interest of efficiency, for each ink we printed all CPWs first, then we replaced the 100 µm nozzle with a 300 µm nozzle and printed all of the accompanying 4PP pads. To calibrate deposition rates, we employed the inkwell method as described by Gu *et al.* [1] wherein the operator modulates the AJP flow rates until deep reactive ion etched inkwells of known and precise volumes are filled over a prescribed time interval. Key parameters associated with printing features using each of the three inks are shown in Table 7. Representative toolpath files (“.prg”) and additional print details (“Deposition Details <ink>.txt”) are included in the file repository.

The total average print time required for each sub-sample is approximately 20 min. Based on this, the nominal time required to print all 15 sub-samples for a given ink is approximately 300 min, or 5 h. However, this is a nominal print time and doesn't include any of the overhead time associated with setting up the system, calibrating deposition rates, switching substrates, updating tool path files, etc. As it turns out, we required a minimum of two working days to print all sub-samples for a given ink. Upon factoring in issues such as equipment scheduling, weekends, and unanticipated setbacks, complete printing of all 45 samples required approximately 11 calendar days. Additionally, sintering samples in a programmable vacuum oven required upwards of eight hours per sample (details below) resulting in a maximum throughput of two substrates per day. Considering the sum total time required to 1) print samples from each of the three inks on all substrates and 2) sinter each substrate using distinct sintering conditions, it was important for us to strategically consider the order in which we printed using each ink and storage conditions for un-sintered samples.

Based on the results of the storage study (below), we chose to simply store incomplete samples in petri dishes in air (Fig. 1b). Furthermore, we chose to print samples in order of apparent decreasing ink stability (Fig. 7) where we printed using Clariant ink first, followed by ANP ink, and finally using UT Dots ink. Due to the previously discussed printing and sintering time requirements, samples were stored for durations ranging from 2 days to 27 days prior to sintering (Table 1-Table 3).

For this study, we investigated the effects of sintering at temperatures of 145 °C, 165 °C, 185 °C, 205 °C, and 225 °C, and in atmospheres of air, nitrogen, and vacuum (Fig. 9). We deposited one sub-sample from each of the three inks onto each of fifteen prepared sample substrates. In this way, we ensured that sintering conditions were identical across all inks (Fig. 1a). We sintered all samples in a Fisher Scientific Isotemp 282A programmable vacuum oven. For sintering in vacuum, the oven chamber was evacuated for 30 minutes prior to ramping the chamber temperature. Ultimate vacuum pressures were approximately 60 Torr. For sintering in nitrogen, we employed a manual flow controller to modulate the flow of dry nitrogen gas (from a LN2 source) to the repressurization inlet of the oven. Prior to sintering, the vacuum chamber was purged with a high flow of nitrogen for 10 min followed by a constant flow rate of 9 SLPM during sintering.

Temperatures were measured using an Omega Type K thermocouple that we positioned directly below the samples near the center of the oven chamber. The thermocouple was connected to a Keithley 2400 Source Meter as was a calibrated MF52C1103F3380 thermistor that was affixed to the thermocouple's cold junction in order to correct the thermocouple readings. We wrote a custom LabVIEW virtual instrument (VI) that read in, corrected, and logged all temperature values. A copy of this VI is included in the file repository. The Logged temperature data is available in raw form in the repository as “.lvm” files. To achieve desirable sintering temperature profiles where temperature overshoot was avoided, we opted to bypass the oven's PID control by creating our own custom sintering programs. An example program schematic is shown in Fig. 10a where an ultimate sintering temperature of 225 °C for 30 min was targeted. Actual sintering temperature profiles are shown in Fig. 10b. In all cases, after holding the target temperature for 30 min, we left the oven to passively cool for approximately 5 h (< 75 °C) before removing the samples.

After sintering, we measured the sheet resistance of each 8 mm x 3 mm pad using a co-linear 4-Point Probe (4PP) setup. We employed a Signatone SP4-40045TBJ 4PP head which has a pin spacing of 40 mil, 10 mil radius tungsten carbide tips, and 45 g spring pressure. The pins were centered over each respective pad and lowered until the springs were fully engaged using a Signatone S-725SRM micropositioner. The 4PP head was connected to a Keithley 2400 Source Meter which we used to sweep the voltage in 0.1 V increments from -1.0 V to +1.0 V across the center pins and measure the resulting current through the outer pins. The source meter was controlled through a LabVIEW VI which calculated the sheet resistance based on the slope of a line fitted to the current vs. voltage data and an appropriate correction factor based on the sample geometry [2]. For our 8 mm x 3 mm samples, we determined the appropriate correction factor to be 2.69. A copy of this LabVIEW VI is also included in the file repository. Three sheet resistance measurements were taken for each sub-sample. Between each measurement the 4PP head was raised, the sample position adjusted, and the head lowered again.

The 4PP pad thicknesses were measured using a Bruker Dektak XT Profiler fitted with a 2 µm stylus. To adequately survey the pads, we employed the profiler's 3D mapping function wherein multiple 2D profiles (Fig. 11a) are obtained at regular intervals and combined to form three dimensional representations of a sample's height over a given area (Fig. 11b). In this way, we were able to acquire more statistically meaningful film thickness values for calculating each sub-sample's conductivity. Each individual scan was taken using a scan rate of approximately 71 µm/s and a minimum stylus force of 0.03 mg was selected to avoid damaging the samples. We performed 3D mapping by obtaining ten 2D profiles with an inter-profile spacing of 700 µm.

The radio frequency measurements were taken using an on-wafer manual/semi-automated microwave probe station from Cascade in ambient air, using a Keysight E8364B PNA network analyzer with a frequency range of 10 MHz to 50 GHz. Additionally, 250 micron pitch Cascade Air Coplanar Probes (ACP) were used to measure the S parameters. Before taking measurements, we calibrated the system using an on-wafer Line-Reflect-Reflect-Match (LRRM) calibration substrate over the range of 10 MHz to 40 GHz. The data was recorded in standard S2P format with nine columns of data. The first column is the measurement frequency, and the remaining 8 are divided into 4 vector pairs for the magnitude and phase angle, respectively, for S11, S21, S12, and S22. Prior to plotting, we converted the S21 magnitude values to decibels using Eq. 1.

(1) $$S21_{dB}=20\log_{10}\left(S21_{mag}\right)$$

### 2.2 Storage study

For this work, we chose to deposit on 2” × 3” Soda Lime Glass 0215 Corning Glass Slides, each of which we cleaned manually using Micro-90 detergent, followed by rinsing with distilled water, drying with compressed air using a blow gun, and treating for 10 min in an 18 Watt air plasma (Harrick PDC-32G) at approximately 100 mTorr. Printing was carried out using an Optomec Aerosol Jet Deposition System (AJ300-UP) with a Sprint UA Max Ultrasonic Atomizer to produce the aerosol. We investigated the same three commercially available silver nano-particle inks as in the sintering study; ANP Silverjet DGP 40LT-15C (‘ANP’), Clariant Prelect TPS 50 G2 (‘Clariant’), and UT Dots UTDAg40x (‘UT Dots’). Prior to loading the inks into the printer, we chose to dilute them, as appropriate, to optimize their printability. Here, we used the same recipes and quantities as outlined above in the sintering study.

Since this study was limited to DC conductivity analysis only, it was only necessary to print samples for 4­Point Probe (4PP) measurements. Hence, for each sample, we deposited an 8 mm x 3 mm pad; the same geometry as the 4PP pads used in the sintering study. Print conditions matched those for 4PP pads outlined in Table 7, and in the “Deposition Details” files from the sintering study. We printed fifteen samples using each ink, five samples on each of three substrates (a total of nine substrates). To avoid any bias resulting from print order, we implemented a print traversal scheme that printed each sample sequentially over all three substrates (Fig. 6).

We sintered each of the substrates using either a Fisher Isotemp 281 oven or a Fisher Isotemp 282A oven at standard atmospheric pressure in air. We manually recorded the chamber temperature immediately prior to inserting samples and immediately prior to removing them using an Omega K-type thermocouple probe connected to a Cole-Parmer Digi-Sense Digital Thermometer (Model 8528-40). The thermocouple probe was positioned just below the samples near the center of the chamber. The chamber was pre-heated to approximately 165 °C for ANP, 190 °C for Clariant, and 175 °C for UT Dots. After printing each set of 15 samples using each ink, we immediately sintered 1 of the 3 sets of 5 samples, “SUBSTRATE A”, for 1 h. Additionally, for each ink, we stored the second set of 5 samples, “SUBSTRATE B”, for 8 days in a closed disposable petri dish in air prior to sintering and the third set, “SUBSTRATE C”, was stored for 8 days under vacuum (∼60 Torr).

After sintering, we measured the sheet resistance of each 8 mm × 3 mm pad using a co-linear 4PP setup. We employed a Signatone SP4-40045TBJ 4PP head which has a pin spacing of 40 mil, 10 mil radius tungsten carbide tips, and 45 g spring pressure. The pins were centered over each respective pad and lowered until the springs were fully engaged using a Signatone S-725SRM micropositioner. The 4PP head was connected to a Keithley 2400 Source Meter which we used to sweep the voltage in 0.1 V increments from -1.0 V to +1.0 V across the center pins and measure the resulting current through the outer pins. The source meter was controlled through a LabVIEW Virtual Instrument which calculated the sheet resistance based on the slope of a line fitted to the current vs. voltage data and an appropriate correction factor based on the sample geometry [2].

We measured 4PP pad thicknesses using a Bruker Dektak XT Profiler fitted with a 2 µm stylus. For each sample a single 2D profile was obtained using a scan rate of approximately 71 µm/s and a stylus force of 0.03 mg to avoid damaging the samples. After performing simple 2-point linear tilt corrections, we measured height values using the substrate region of each dataset as a reference point and the average height of the roughly 2.75 mm flat section across the width of the pad. A representative 2D scan is shown in Fig. 11a.

## Declaration of Competing Interest

The authors declare that they have no known competing financial interests or personal relationships which have, or could be perceived to have, influenced the work reported in this article.

## Appendix Supplementary materials

Image, application 1

Image, application 2

Image, application 3
