# Supplementary figures and images for: Conductivity and radio frequency performance data for silver nanoparticle inks deposited via aerosol jet deposition and processed under varying conditions
Source: Data Brief. 2020 Sep 24;33:106331. doi: 10.1016/j.dib.2020.106331 (PMC7560714; doi:10.1016/j.dib.2020.106331)

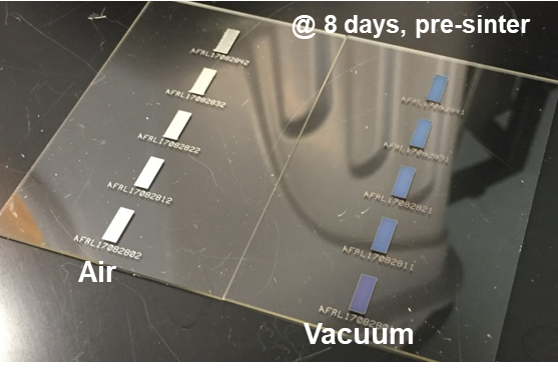

Supplement: Supplementary file 1 [file mmc1.zip › Storage Study/Clariant Ink/pre-sinter.png]

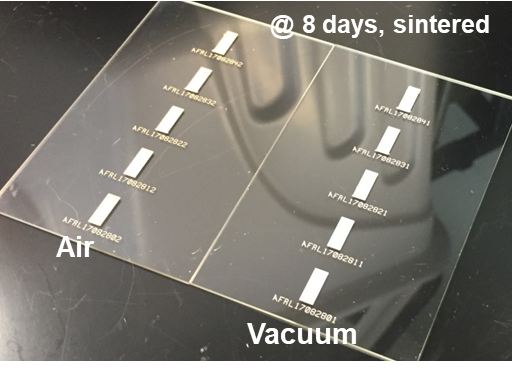

Supplement: Supplementary file 1 [file mmc1.zip › Storage Study/Clariant Ink/sintered.png]
